# Comparative analysis of adsorption and gas-sensing performance in metal oxide-functionalized GaN nanotubes for real-time CF_3_CN detection in GIS high-voltage systems

**DOI:** 10.3389/fchem.2026.1820485

**Published:** 2026-05-22

**Authors:** Xunting Wang, Hongxia Wang, Fuqi Ma

**Affiliations:** 1 Electric Power Research Institute of State Grid Anhui Electric Power Co Ltd, State Grid Anhui Electric Power Co Ltd, Hefei, Anhui, China; 2 Department of Electrical and Computer Engineering, University of Denver, Denver, CO, United States; 3 School of Electrical and Automation, Wuhan University, Wuhan, Hubei, China; 4 School of Electrical Engineering, Xi’an University of Technology, Xi an, Shaanxi, China

**Keywords:** chemical bonding, gas-sensing performance, GIS, HV electrical equipment, real-time detection

## Abstract

The eco-friendly gas C_4_F_7_N has emerged as a promising alternative to SF_6_ for online monitoring in gas-insulated switchgear (GIS) high-voltage (HV) electrical systems, but partial discharges-an inherent issue in such environments-cause C_4_F_7_N decomposition, generating byproducts like CF_3_CN. This study employs first-principles calculations to systematically investigate the adsorption and electronic properties of metal oxide-modified GaN nanotubes (GaNNTs) (CuO, ZnO, Ag_2_O, CrO_2_) toward CF_3_CN, analyzing key parameters such as band gaps, density of states (DOS), differential charge density (DCD), and molecular orbital interactions. The results demonstrate that metal oxide modification enhances the conductivity of GaNNTs by significantly reducing their band gaps compared to pristine GaNNTs, with decreases of 40.13% (CuO), 67.97% (ZnO), 43.59% (Ag_2_O), and 49.91% (CrO_2_). For CF_3_CN adsorption, CuO-GaNNT exhibits an adsorption energy of −0.399 eV and a distance of 2.584 Å, Ag_2_O-GaNNT shows −0.746 eV and 2.120 Å, CrO_2_-GaNNT displays −0.243 eV and 3.040 Å, while ZnO-GaNNT undergoes chemical adsorption with a higher energy of −2.478 eV and a shorter distance of 1.319 Å. The adsorption capacity follows the order ZnO-GaNNT > Ag_2_O-GaNNT > CuO-GaNNT > CrO_2_-GaNNT. Regarding recovery at room temperature, Ag_2_O-GaNNT, CuO-GaNNT, and CrO_2_-GaNNT demonstrate favorable desorption behavior, whereas ZnO-GaNNT exhibits slower recovery due to stronger chemical bonding. These computational findings highlight the potential of metal oxide-modified GaNNTs as advanced materials for real-time detection of C_4_F_7_N and mitigation of its decomposition product, CF_3_CN, in GIS HV equipment monitoring.

## 1 Introduction

Globally, there is a rapidly accelerating shift toward green, low-carbon, and environmentally sustainable development, driving an urgent demand for high-performance, eco-friendly insulation materials in HV electrical systems, including GIS and transformers (Salama et al., 2020; Sang et al., 2023; Zhu and Zhou, 2021). For decades, sulfur hexafluoride (SF_6_) has been the dominant insulation medium due to its superior electrical properties (Yang et al., 2021). In recent studies, many researchers have conducted extensive research on the decomposition products and adsorption properties of SF_6_. S. Mejía Sintillo et al. studied Cu_n_ Clusters (n = 13, 43, and 55) as Possible Degradant Agents of mSF_6_ Molecules (m = 1, 2), they found the electronic gaps do not exhibit drastic changes after adsorption of mSF_6_ molecules, and the magnetic moment remains without alterations (Sintillo et al., 2022). However, its extremely high global warming potential (GWP) and the detrimental environmental impacts of leaks have prompted international concerns, compelling the power industry to urgently seek low-GWP, environmentally benign alternatives. Among these, C_4_F_7_N has emerged as a leading candidate, praised for its comparable electrical performance to SF_6_, significantly lower GWP, and exceptional chemical stability (Oche et al., 2023; Oyo-Ita et al., 2024). Nevertheless, under operational stress conditions such as partial discharges or overheating in HV equipment, C_4_F_7_N can decompose, generating CF_3_CN gas (Ogungbemiro et al., 2023; Parambath et al., 2023). Effective monitoring of CF_3_CN is therefore critical, enabling early fault detection in GIS and transformers, timely warnings, and the prevention of catastrophic failures like large-scale power outages.

Currently, the dominant methods for CF_3_CN detection, such as gas chromatography (Chen et al., 2021; Cui et al., 2019) and mass spectrometry (Li et al., 2018; Zhong et al., 2019), offer high accuracy but are hindered by complex operational requirements and lengthy analysis times, making them impractical for real-time, on-site monitoring (Chachereau et al., 2018). Thus, there is an urgent need for a rapid, simple, and precise CF_3_CN detection technology to facilitate the broader adoption of C_4_F_7_N in electrical systems. Nanomaterials, renowned for their unique physicochemical properties, are widely utilized in gas-sensing applications (Chen et al., 2020; Xu et al., 2022; Zhao et al., 2022). Among them, gallium nitride nanotubes (GaNNTs)-emerging wide-bandgap semiconductors-show exceptional promise due to their superior conductivity, thermal stability, and chemical robustness (Is et al., 2022; Wang et al., 2019). Surface functionalization has been demonstrated to significantly enhance the gas-sensing performance of two-dimensional materials. For example, Oyo-Ita et al. improved the ethanol adsorption capacity of GaNNTs by incorporating S, P, and Si atoms (Gui et al., 2019), while Oche et al. reported enhanced surface reactivity for dissolved gas detection (C_2_H_2_, CH_4_, H_2_) in oil through Re and Tc doping (Gui et al., 2020). Similarly, Ogungbemiro et al. found that transition metal doping (Cu, Ag, Au) increased the sensitivity of GaNNTs (Wang et al., 2019), and Chen et al. observed a marked boost in surface reactivity with Pd doping (Grimme et al., 2010). Metal oxides, as metallic derivatives, also exhibit strong catalytic and modification capabilities. Xu et al. demonstrated that integrating metal oxide particles (ZnO, CuO) elevated the Fermi level in graphene, improving its surface conductivity (Pack and Monkhorst, 1977). Chen et al. further reported that SnS_2_ modified with metal oxides (CuO, NiO) displayed high sensitivity to dissolved gases in oil (C_2_H_2_, CH_4_, H_2_, H_2_O) (Zheng et al., 2023). Despite these advancements, research on metal oxide-modified GaNNTs for gas sensing remains limited, highlighting a critical gap in the development of next-generation CF_3_CN detection technologies.

Building on existing research, this study utilizes density functional theory (DFT) to systematically investigate the adsorption behavior of pristine GaNNTs and metal oxide-doped GaNNTs (CuO, ZnO, Ag_2_O, CrO_2_) toward CF_3_CN. The analysis focuses on key parameters, including charge transfer dynamics, adsorption energy, DOS, and DCD, to elucidate the interaction mechanisms. Furthermore, the feasibility of these doped GaNNTs as CF_3_CN sensors is evaluated, with the aim of providing a robust scientific basis for the safe implementation of C_4_F_7_N in HV electrical equipment. The findings are expected to support the power industry’s shift toward sustainable practices while advancing the development of high-performance CF_3_CN sensing technologies.

## 2 Computational details

This study employs DFT (Chu et al., 2023; Fá et al., 2023; Wang et al., 2020) within the DMol^3^ module of Materials Studio to perform optimization calculations. To model individual doping and adsorption scenarios, a large GaN nanotube (GaNNT) supercell (48 Ga and 48 N atoms) is constructed within a 25 × 25 × 13 Å vacuum region. The electron exchange-correlation interaction is treated using the Perdew–Burke–Ernzerhof (PBE) functional (Epping and Koch, 2023) under the generalized gradient approximation (GGA) (Jayathilaka et al., 2019), with Grimme’s dispersion correction included to account for long-range van der Waals forces. A double numeric polarized (DNP) basis set (Baig et al., 2019; Shokri and Salami, 2016) is adopted for enhanced accuracy. For structural and energy optimizations, a 1 × 1 × 5 Monkhorst-Pack k-point grid (Yin et al., 2016) is used for Brillouin zone sampling, with convergence thresholds set to 1 × 10^−6^ Ha (energy), 5 × 10^−3^ Ha/Å (maximum displacement), and 2 × 10^−3^ Å (maximum force) (Kanoun et al., 2022). Self-consistent field (SCF) calculations employ an energy convergence criterion of 10^−6^ Ha and a global orbital cutoff radius of 5.0 Å (Tan et al., 2024). To improve electronic DOS resolution near the Fermi level, an orbital smearing value of 5 × 10^−3^ Ha is applied (Chen J. et al., 2023).

For the purpose of assessing the properties of the doping system, the doping energy (*E*_b_) is computed by employing the following Equation 1:

$$E_{b}=E_{\text{MO}‐\text{GaNNT}}\;‐\;E_{\text{MO}}\;‐\;E_{\text{GaNNT}}$$ (1)

where the *E*_MO-GaNNT_, *E*_MO_, *E*_GaNNT_ are the total energy of MO-GaNNT (MO = CuO, ZnO, Ag_2_O, CrO_2_), isolated MO (CuO, ZnO, Ag_2_O, CrO_2_) and pure GaNNT, respectively.

For the adsorption system, the adsorption energy (*E*_abs_) is characterized through the Equation 2:

$$E_{\text{ads}}=E_{\text{CF}3\text{CN}/\text{MO}‐\text{GaNNT}}\;‐\;E_{\text{MO}‐\text{GaNNT}}\;–\;E_{\text{CF}3\text{CN}}$$ (2)

where the *E*_CF3CN/MO-GaNNT_, *E*_MO-GaNNT_, *E*_CF3CN_ are the total energy of the adsorbed systems CF_3_CN/MO-GaNNT (MO = CuO, ZnO, Ag_2_O, CrO_2_), the modified system MO-GaNNT (MO = CuO, ZnO, Ag_2_O, CrO_2_) and CF_3_CN gas molecule, respectively.

Throughout the doping and adsorption procedures, charge transfer (denoted as *Q*_t_) takes place. This charge transfer value can be determined by employing Mulliken population analysis. The precise formula for calculating *Q*_t_ is presented in Equation 3:

$$Q_{t}=Q_{1}‐Q_{2}$$ (3)

where *Q*_2_ and *Q*_1_ are the total charge of CF_3_CN before and after adsorption, respectively.

Equation 4 furnishes the mathematical expression for the energy gap (*E*_g_) that exists between the lowest unoccupied molecular orbital (LUMO) and the highest occupied molecular orbital (HOMO):

$$E_{g}=|E_{\text{LUMO}}\;‐E_{\text{HOMO}}|$$ (4)

where the *E*_HOMO_ and *E*_LUMO_ are the energy of HOMO and LUMO, respectively.

## 3 Results and discussion

### 3.1 Analysis of geometric and electronic traits of metal oxide-modified GaNNTs post CF_3_CN adsorption

**FIGURE 1:**
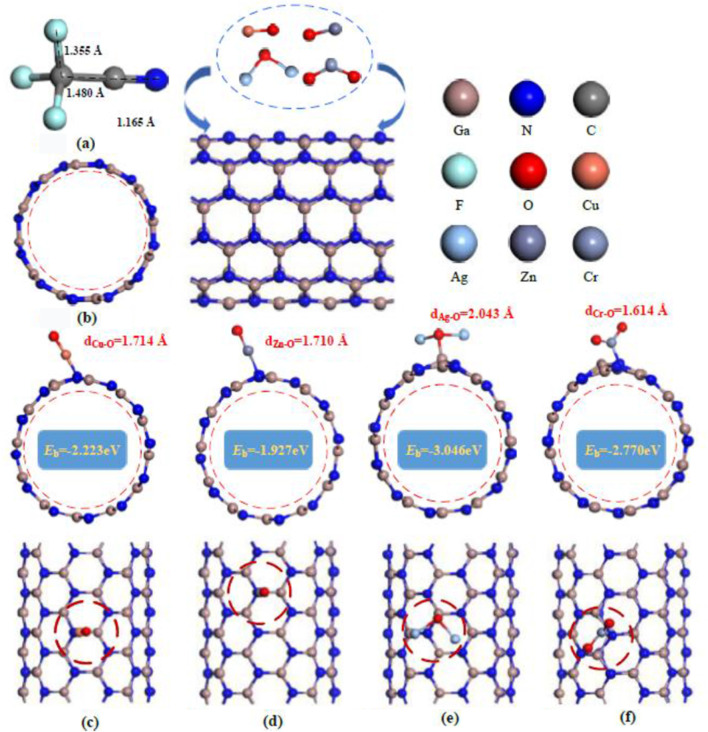
The most stable configurations of metal oxide-modified GaNNTs interacting with the target gas (the distance between atoms is shown in the figure, d, Å). **(a)** CF_3_CN **(b)** CuO doped GaNNTs **(c)** ZnO doped GaNNTs **(d)** Ag_2_O doped GaNNTs **(e)** CrO_2_ doped GaNNT.

Figure 1 illustrates the optimized structures of CF_3_CN and GaNNTs, both pristine and modified with metal oxides (CuO, ZnO, Ag_2_O, CrO_2_). Figure 1a shows the CF_3_CN molecule, featuring a central carbon atom covalently bonded to three fluorine atoms and a cyano group (C≡N), with key bond lengths of 1.355 Å (C–F), 1.480 Å (C–C), and 1.165 Å (C≡N) reflecting its polar nature. Figure 1b depicts the pristine GaN nanotube (GaNNT), which exhibits a hexagonal lattice composed of alternating gallium (Ga) and nitrogen (N) atoms, forming a cylindrical geometry with a high surface area ideal for gas-sensing applications; the metal–oxygen bond lengths in the modifying metal oxides are 1.714 Å (CuO), 1.710 Å (ZnO), 2.043 Å (Ag_2_O), and 1.614 Å (CrO_2_), indicating variations in bonding strength. Figures 1c–f demonstrate that GaNNTs modified with CuO and ZnO retain a circular cross-section, suggesting structural stability post-modification, while Ag_2_O and CrO_2_ modifications exhibit distinct morphological changes.

In comparison, doping with Ag_2_O and CrO_2_ leads to minor bending deformations in the GaNNTs; however, the overall tubular structure remains intact, showcasing their excellent structural resilience. The computed binding energies for the modified GaNNTs are as follows: 2.223 eV for CuO-doped GaNNT, −1.927 eV for ZnO-doped GaNNT, −3.046 eV for Ag_2_O-doped GaNNT, and −2.770 eV for CrO_2_-doped GaNNT. Regarding doping distances, the sequence is: ZnO-GaNNT (1.381 Å) < CrO_2_-GaNNT (1.793 Å) < Ag_2_O-GaNNT (1.899 Å) < CuO-GaNNT (1.924 Å), with all values falling below 2 Å. This confirms the remarkable stability of the doped GaNNT structures.

**FIGURE 2:**
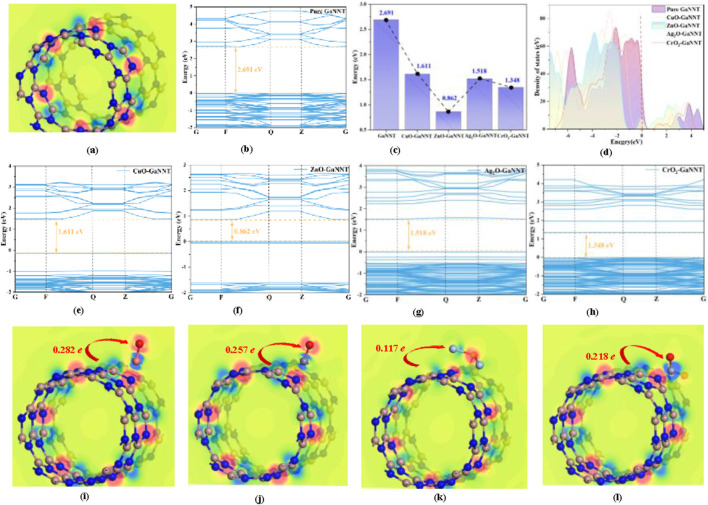
Electrical characteristics of metal oxide-modified GaNNTs for sensing applications. **(a–i)** The names of the band structures for different systems are shown in the figures, and the vertical axis represents different energy levels.

Figure 2 offers a comprehensive analysis of the electronic transition properties of GaNNTs in both pristine and metal oxide-doped (CuO, ZnO, Ag_2_O, CrO_2_) states, revealing that the unmodified GaNNT exhibits a band gap of 2.691 eV, which narrows significantly upon doping to 1.611 eV (CuO), 0.862 eV (ZnO), 1.518 eV (Ag_2_O), and 1.348 eV (CrO_2_), corresponding to reductions of 40.13%, 67.97%, 43.59%, and 49.91%, respectively, indicating enhanced electrical conductivity in the modified systems; band structure comparisons in Figures 2e–h demonstrate pronounced electronic reorganization, particularly in Ag_2_O-GaNNT and CrO_2_-GaNNT, with increased DOS near the valence band maximum and a conduction band minimum closer to the Fermi level, reflecting improved semiconductor characteristics; the total DOS (TDOS) in Figure 2d shows new energy peaks introduced by doping within the range of approximately −4 eV to −2 eV, while all doped systems exhibit a leftward shift relative to pristine GaNNT, consistent with band gap narrowing—most notably, ZnO-GaNNT shifts by ∼2.2 eV, aligning with its largest band gap reduction; DCD plots in Figures 2i–l reveal electron redistribution during doping, with red and blue regions indicating accumulation and depletion, respectively, and charge transfer analysis confirms that GaNNT donates 0.282 e, 0.257 e, 0.117 e, and 0.218 e to CuO, ZnO, Ag_2_O, and CrO_2_, respectively, underscoring the strong electronegativity and electron-accepting capacity of the metal oxides, with detailed doping parameters summarized in Table 1.

**TABLE 1:** The *E*_b_, *Q*_t_, and doping distance (*d*) on MO-GaNNTs.

Doping system	*E*_b_ (eV)	*Q*_t_ (*e*)	*d* (Å)
CuO-GaNNT	−2.223	−0.282	1.924 (Cu-N)
ZnO-GaNNT	−1.927	−0.257	1.381 (Zn-N)
Ag_2_O-GaNNT	−3.046	−0.117	1.899 (O-Ga)
CrO_2_-GaNNT	−2.770	−0.218	1.793 (Cr-N)

### 3.2 Electronic properties and adsorption characteristics of MO-modified GaNNTs upon CF_3_CN adsorption

**FIGURE 3:**
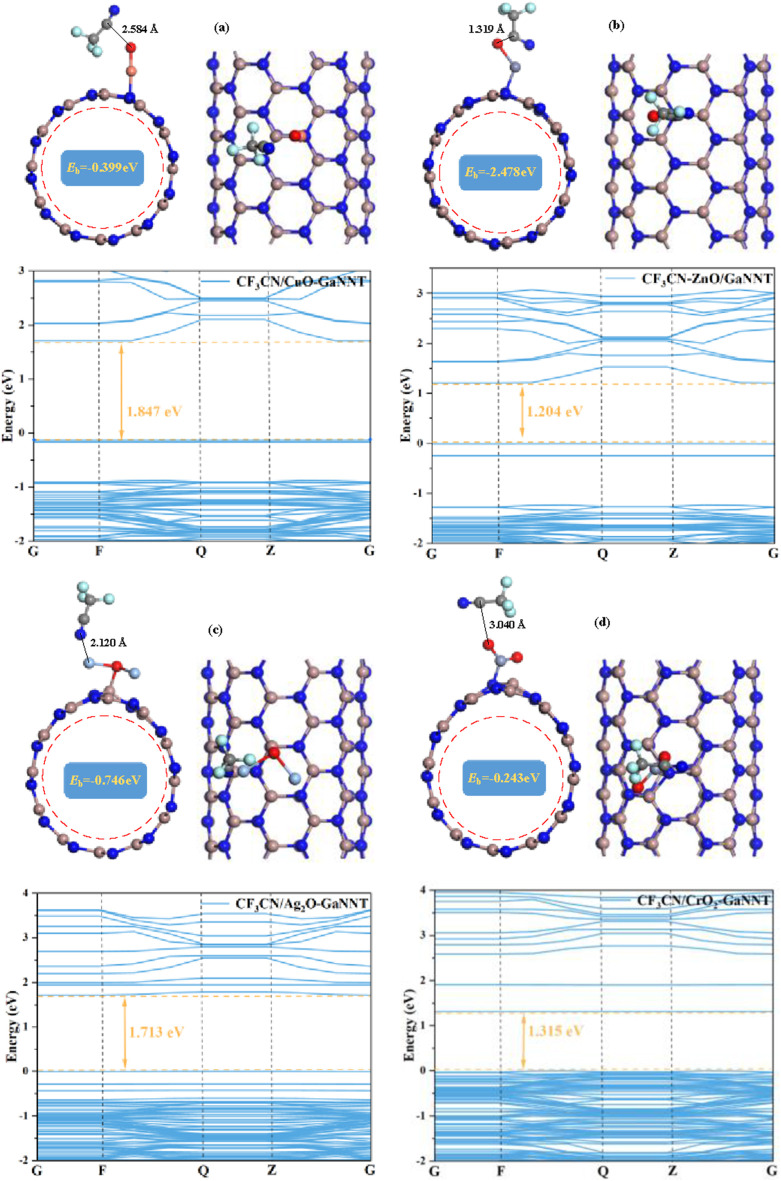
The most stable adsorption configuration of CF_3_CN on MO-modified GaNNTs and its resulting band structure **(a)** CF_3_CN/CuO-GaNNTs **(b)** CF_3_CN/ZnO-GaNNTs **(c)** CF_3_CN/Ag_2_O-GaNNTs **(d)** CF_3_CN/CrO_2_-GaNNTs.

Figure 3 presents the most stable configurations of CuO-, ZnO-, Ag_2_O-, and CrO_2_-modified GaNNTs (MO–GaNNTs) following CF_3_CN adsorption, along with corresponding band structure diagrams. When combined with structural observations from Figures 1c–f, it becomes evident that MO–GaNNTs retain stable tubular architectures without noticeable morphological changes after CF_3_CN adsorption, highlighting their exceptional structural robustness. Detailed computational results reveal adsorption energies of −0.399 eV for CF_3_CN/CuO–GaNNT, −2.748 eV for CF_3_CN/ZnO–GaNNT, −0.746 eV for CF_3_CN/Ag_2_O–GaNNT, and −0.243 eV for CF_3_CN/CrO_2_–GaNNT. The negative values signify exothermic adsorption processes, confirming strong interactions between the adsorbent and gas molecules, as well as stable gas attachment on MO–GaNNT surfaces. Adsorption distances are measured at 2.584 Å (CF_3_CN/CuO–GaNNT), 1.319 Å (CF_3_CN/ZnO–GaNNT), 2.120 Å (CF_3_CN/Ag_2_O–GaNNT), and 3.040 Å (CF_3_CN/CrO_2_–GaNNT), suggesting chemical adsorption for ZnO–GaNNT and physical adsorption for the other three systems. Based on both adsorption energy and distance metrics, the adsorption efficiency follows the order: ZnO–GaNNT > Ag_2_O–GaNNT > CuO–GaNNT > CrO_2_–GaNNT. Notably, ZnO–GaNNT exhibits a significant advantage, with an adsorption energy 11.31 times greater and an adsorption distance 56.6% shorter than those of CrO_2_–GaNNT, underscoring its superior adsorption performance. Comparative analysis of the original band structures in Figures 2e–h with post-adsorption results in Figure 3 reveals notable changes in band width: CF_3_CN/CuO–GaNNT increases to 1.847 eV (a 14.6% rise), CF_3_CN/ZnO–GaNNT to 1.204 eV (a 39.7% rise), and CF_3_CN/Ag_2_O–GaNNT to 1.713 eV (a 12.8% rise), while CF_3_CN/CrO_2_–GaNNT narrows from 1.348 eV to 1.315 eV. Broader band widths typically correlate with reduced conductivity, whereas narrower ones suggest enhanced conductivity; both scenarios can significantly alter electrical signals in sensor applications. These findings confirm the sensitivity and responsiveness of CuO-, ZnO-, Ag_2_O-, and CrO_2_-doped GaNNTs to CF_3_CN adsorption, making them promising candidates for gas detection systems.

**FIGURE 4:**
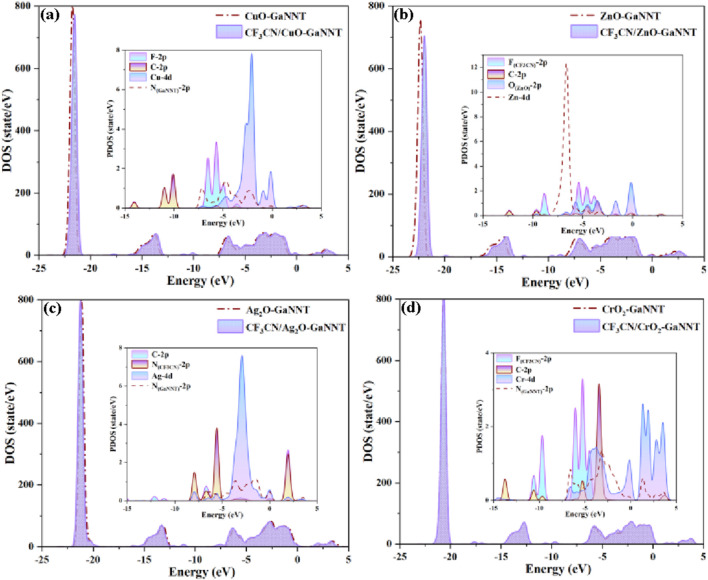
The TDOS and PDOS for all adsorption systems. **(a–d)** The interpretations of the different molecular orbitals are shown in the figures.

To systematically explore the electronic interactions during CF_3_CN adsorption on metal oxide (MO)-modified GaNNT composites, Figure 4 presents a comparative analysis of the TDOS for MO (CuO, ZnO, Ag_2_O, CrO_2_)–GaNNT systems before and after adsorption, complemented by partial DOS (PDOS) plots in the insets; in the CuO–GaNNT system (Figure 4a), the post-adsorption DOS shows a slight rightward shift with a new peak emerging near −10 eV, which PDOS analysis attributes primarily to interactions between the C-2p orbitals of CF_3_CN and adjacent atomic orbitals, while Cu-4p orbital hybridization significantly enhances electronic states near the Fermi level, indicating localized electronic restructuring; for the CF_3_CN/ZnO–GaNNT system, a more pronounced rightward shift in the DOS after adsorption suggests increased electron occupancy within the band gap, strongly supporting a chemical adsorption mechanism involving stable bond formation between CF_3_CN and ZnO–GaNNT, with PDOS analysis highlighting highly active states from F-2p, C-2p, and O-2p orbitals in the −6.2 eV to −2.5 eV range, demonstrating strong orbital hybridization and the substantial influence of CF_3_CN interaction on the electronic structure; in contrast, the Ag_2_O–GaNNT system exhibits a leftward shift in the DOS curve following CF_3_CN adsorption, reflecting altered electron filling states, with major contributions below the Fermi level originating from N-2p orbitals of CF_3_CN, Ag-4d orbitals, and N-2p orbitals of Ag_2_O–GaNNT, while above the Fermi level, hybridization involves N-2p (CF_3_CN) and C-2p orbitals; notably, the CrO_2_–GaNNT system shows minimal changes in the total DOS after CF_3_CN adsorption, consistent with physical adsorption behavior, yet PDOS analysis reveals significant overlap and crossing of F-2p, C-2p, Cr-4d, and N-2p energy levels, indicating orbital hybridization even without chemical bond formation, with F-2p and C-2p orbitals contributing prominently below the Fermi level and Cr-4d orbitals dominating above it; these findings provide detailed insights into the electronic mechanisms governing CF_3_CN adsorption on MO–GaNNT composites, emphasizing the distinct roles of chemical versus physical interactions in shaping their electronic properties.

**FIGURE 5:**
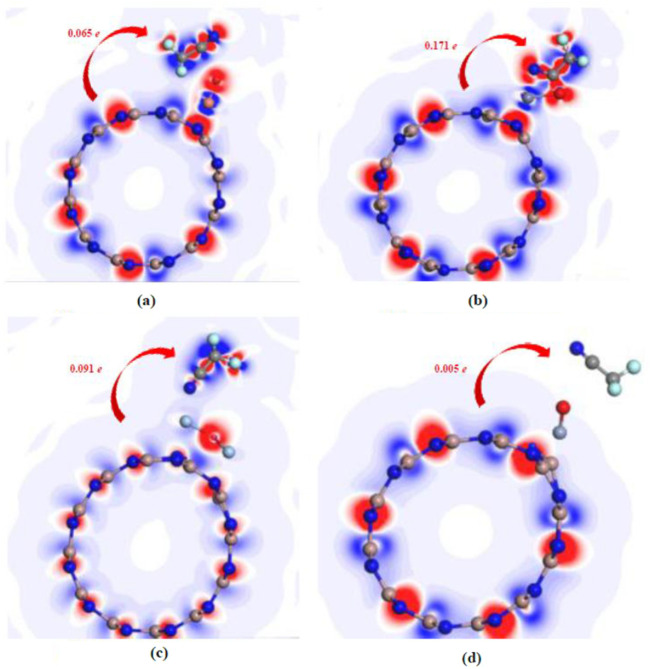
DCD distribution for each adsorption systems. **(a–d)** The interpretations of the different molecular orbitals are shown in the figures.

Figure 5 displays the DCD distributions, providing a vivid depiction of electron transfer dynamics across various adsorption systems interacting with CF_3_CN, where red regions signify electron accumulation (increased electron density) and blue regions indicate electron depletion; electron transfer is clearly evident in all four adsorption configurations, revealing the redistribution and migration of electrons during the process; a key observation is that metal oxide-modified GaNNTs (MO–GaNNTs) function as electron donors when interacting withCF_3_CN, with the extent of electron donation quantified by the number of electrons transferred to CF_3_CN: 0.065 e for CF_3_CN/CuO–GaNNT, 0.171 e for CF_3_CN/ZnO–GaNNT, 0.091 e for CF_3_CN/Ag_2_O–GaNNT, and 0.005 e for CF_3_CN/CrO_2_–GaNNT; these variations highlight the influence of different metal oxide modifications on electron transfer efficiency, underscoring the strong link between material composition and electronic behavior; the DCD maps offer deeper insights into charge redistribution between CF_3_CN and the adsorbent substrates, such as the intense red region in the CF_3_CN/ZnO–GaNNT system confirming substantial electron gain by CF_3_CN, reflecting its strong electron-accepting capacity, while the weaker coloration in the CF_3_CN/CrO_2_–GaNNT system indicates limited electron transfer, demonstrating the variability in electron transfer capacity across different adsorption systems; to comprehensively assess the performance of these systems, Table 2 summarizes the corresponding adsorption parameters for each configuration.

**TABLE 2:** The *E*_abs_, *Q*_t_, and adsorption distance (*d*) on MO-GaNNT.

Adsorption system	*E*_abs_ (eV)	*Q*_t_ (*e*)	*d* (Å)
CF_3_CN/CuO-GaNNT	−0.399	−0.065	2.584
CF_3_CN/ZnO-GaNNT	−2.478	−0.171	1.319
CF_3_CN/Ag_2_O-GaNNT	−0.746	−0.091	2.120
CF_3_CN/CrO_2_-GaNNT	−0.243	−0.005	3.040

### 3.3 The molecular orbital theory analysis of doped and adsorbed systems

**FIGURE 6:**
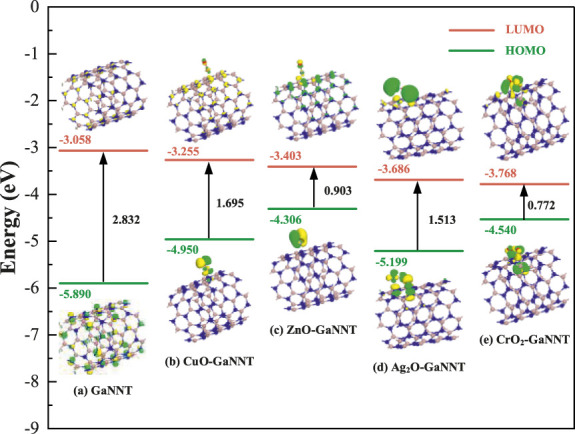
Comparison of HOMO and LUMO distribution before and after doping.

**FIGURE 7:**
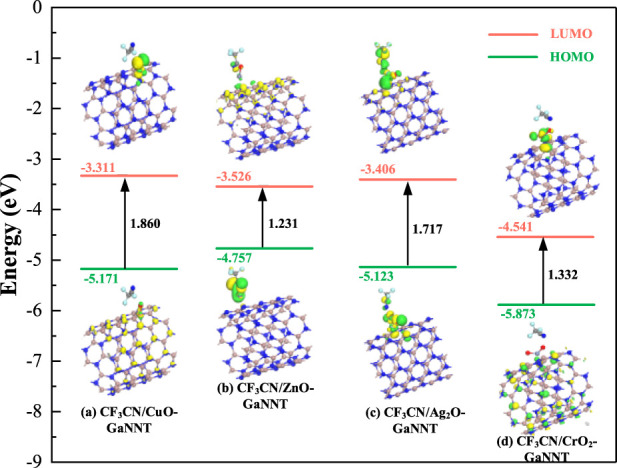
Comparison of HOMO and LUMO distribution of CF3CN/MO-GANNTs.

Figures 6, 7 depict the spatial distributions of the HOMO and LUMO in doped systems both before and after CF_3_CN adsorption, where the HOMO energy level indicates a molecule’s propensity to donate electrons—higher values corresponding to easier electron loss-while the LUMO energy level reflects its capacity to accept electrons, with lower values denoting stronger electron-accepting ability; the energy difference between HOMO and LUMO, known as the HOMO-LUMO gap, is a crucial parameter for evaluating the energy required for electron excitation, with a smaller gap enabling easier electronic transitions; as shown in Figure 5, prior to modification with metal oxides (CuO, ZnO, Ag_2_O, CrO_2_), the HOMO and LUMO of pristine GaNNT are primarily localized on nitrogen atoms, exhibiting a band gap of 2.832 eV, indicating that significant energy is needed to excite electrons in the undoped system; following metal oxide doping, notable changes occur in the electronic structure, particularly a marked reduction in the HOMO-LUMO gap, with measured gaps of 1.695 eV for CuO-doped GaNNT, 0.903 eV for ZnO-doped GaNNT, 1.513 eV for Ag_2_O-doped GaNNT, and 0.772 eV for CrO_2_-doped GaNNT, representing narrowing percentages ranging from 41.1% to 72.7%, which facilitates electron transitions from HOMO to LUMO and enhances electrical conductivity; additionally, after doping, the HOMO density tends to concentrate around the metal oxide sites, while the LUMO accumulates in the doped regions of the GaNNTs, indicating that metal oxide incorporation not only adjusts the energy level alignment but also redistributes electron density throughout the system, thereby optimizing its electronic properties.

A deeper analysis of the systems after CF_3_CN adsorption, as shown in Figure 7, reveals that the adsorption process significantly alters the spatial distribution of the LUMO and HOMO orbitals; across the various metal oxide (CuO, ZnO, Ag_2_O, and CrO_2_)-modified GaNNT composite systems, the metal oxide atoms serve as the primary adsorption sites and strongly influence electron redistribution-for instance, in the CF_3_CN/CuO-GaNNT system, the LUMO concentrates at the interface between CF_3_CN and CuO, while the HOMO localizes mainly on the material surface, indicating a specific electron transfer mode; similarly, other composite systems display distinct orbital distribution features: in CF_3_CN/ZnO–GaNNT, the LUMO is predominantly found on the material surface, with the HOMO localizing between ZnO and the gas molecule; in CF_3_CN/Ag_2_O–GaNNT, both HOMO and LUMO are confined to the region between the dopant and CF_3_CN; and in CF_3_CN/CrO_2_-GaNNT, a larger portion of the LUMO shifts toward the CrO_2_ sites; these results demonstrate that modifying the GaNNT surface with metal oxides (CuO, ZnO, Ag_2_O, and CrO_2_) effectively enhances its surface reactivity, significantly boosting its adsorption capacity for CF_3_CN; when assessing changes in electrical conductivity after CF_3_CN adsorption, the ZnO–GaNNT composite shows the most pronounced improvement, followed by CrO_2_-GaNNT, Ag_2_O-GaNNT, and CuO-GaNNT, highlighting the critical role of metal oxide selection in optimizing both adsorption performance and electronic properties.

### 3.4 The evaluation of the gas sensing and desorption performance

The desorption time (recover tome, *t*) of gas molecules from a material’s surface is a key parameter influencing sensor performance. It can be determined using the Arrhenius equation (Chen Q. L. et al., 2023; Sosa et al., 2022), expressed as Equation 5:

$$t=\left(1/v_{0}\right)\;*\;\exp\left(‐E_{\text{abs}}/\text{KT}\right)$$ (5)

**FIGURE 8:**
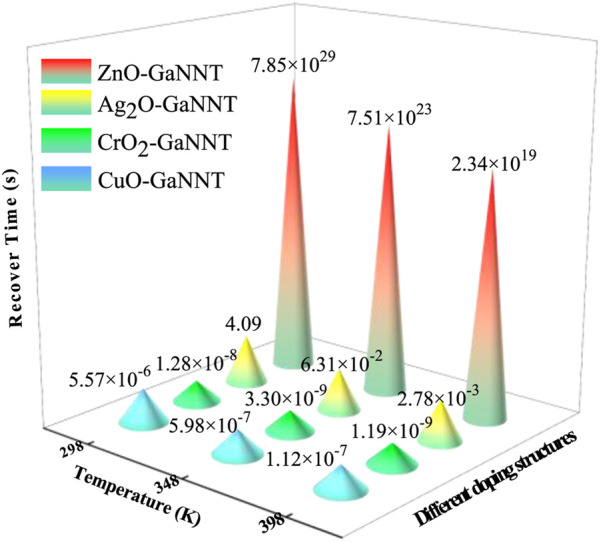
The desorption time (*t*) of CF_3_CN/MO-GaNNT.

In the given equation, *v*_0_ denotes the attempt frequency, generally taken as 10^−12^ s^-1^, which reflects the frequency of collisions between gas molecules and the material surface per unit time; K stands for the Boltzmann constant (8.62 × 10^−5^ eV/K), and T refers to the ambient temperature. In the present work, the desorption time of CF_3_CN was calculated at 298 K (room temperature), 348 K, and 398 K for four different adsorption systems: CF_3_CN/CuO-GaNNT, CF_3_CN/ZnO-GaNNT, CF_3_CN/Ag_2_O-GaNNT, and CF_3_CN/CrO_2_-GaNNT. The corresponding results are presented in Figure 8. For CuO-GaNNT, Ag_2_O-GaNNT, and CrO_2_-GaNNT, which interact with CF_3_CN mainly through physical adsorption, the desorption times at room temperature are 5.57 μs, 4.09 s, and 1.28 × 10^−2^ μs, respectively. These short recovery times indicate the ability of these materials to detect CF_3_CN rapidly. In contrast, ZnO-GaNNT undergoes chemical adsorption with CF_3_CN and exhibits higher adsorption energy, leading to a substantially longer desorption time of 7.85 × 10^29^ s at 298 K. This result highlights its strong affinity and effective retention of CF_3_CN, supporting the suitability of ZnO-GaNNT as an adsorbent for this gas. With increasing temperature, enhanced thermal motion provides molecules with greater kinetic energy to overcome adsorption barriers, thereby shortening the desorption time in all cases. At 398 K, the desorption times from CuO-GaNNT, Ag_2_O-GaNNT, CrO_2_-GaNNT, and ZnO-GaNNT decrease significantly to 1.12 × 10^−7^ s, 2.34 × 10^19^ s, 2.78 × 10^−3^ s, and 1.19 × 10^−9^ s, respectively.

**FIGURE 9:**
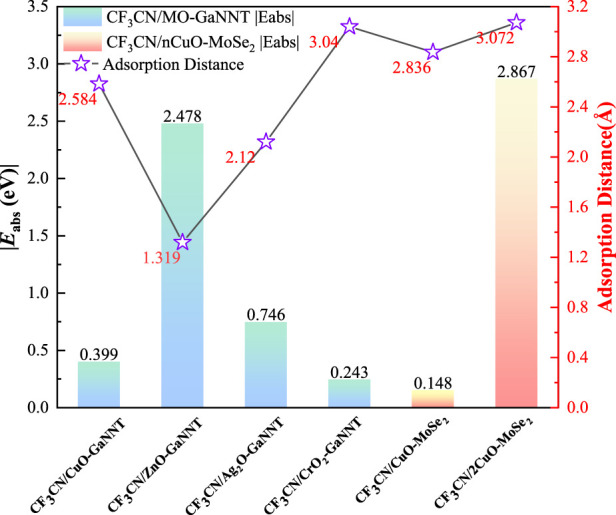
Comparison of adsorption energy and distance for different Materials. The CF_3_CN/nCuO-MoSe_2_ data is sourced from other studies on this new material.

To systematically evaluate the adsorption performance of metal oxide (CuO, ZnO, Ag_2_O, and CrO_2_)-modified GaNNTs toward CF_3_CN, a detailed comparative analysis was performed using benchmark data from the literature (Wang et al., 2021); as shown in Figure 9, the adsorption energies of CF_3_CN on the modified GaNNTs are notably higher than those in reference systems, such as CF_3_CN/CuO-MoSe_2_, which exhibits an adsorption energy of 0.148 eV, highlighting the superior adsorption strength of metal oxide-GaNNT composites for CF_3_CN; beyond the energetic advantages, the adsorption distances in these composite systems are also significantly reduced, with calculated decreases of 8.9% for CuO-GaNNT, 53.5% for ZnO-GaNNT, 25.2% for Ag_2_O-GaNNT, and −7.2% for CrO_2_-GaNNT (indicating a slight increase in this case), demonstrating closer interfacial contact between CF_3_CN and the modified GaNNT surfaces in most configurations; in contrast, while the CF_3_CN/CuO-MoSe_2_ system shows a notably high adsorption energy of 2.867 eV-surpassing that of CF_3_CN/ZnO-GaNNT by 0.389 eV-its adsorption distance increases substantially to 3.072 Å, which is 132.9% longer than that of CF_3_CN/ZnO-GaNNT, suggesting a less favorable geometric arrangement for efficient sensing that may compromise rapid response and recovery characteristics; by evaluating both adsorption energy and distance, it is clear that metal oxide-modified GaNNTs provide a well-balanced and highly effective sensing platform for CF_3_CN detection, as their ability to combine strong adsorption forces with short interaction distances enhances sensor sensitivity and response kinetics, making them promising candidates for advanced sensing applications.

## 4 Conclusion

Based on first-principles computational analysis, this study reveals that metal oxide (CuO, ZnO, Ag_2_O, and CrO_2_)-modified GaNNTs exhibit exceptional effectiveness in adsorbing CF_3_CN, a decomposition byproduct of the eco-friendly SF_6_ alternative gas C_4_F_7_N, with a thorough examination of structural, electronic, and molecular orbital characteristics in both doped and adsorbed states thoroughly elucidating the mechanisms governing CF_3_CN adsorption on MO-GaNNTs; the computational results confirm the superior performance of metal oxide-functionalized GaNNTs in CF_3_CN adsorption, with key findings summarized as follows.

1. Following metal oxide modification, the bandgap of GaNNTs decreases significantly from the original 2.691 eV–1.611 eV (CuO), 0.862 eV (ZnO), 1.518 eV (Ag_2_O), and 1.348 eV (CrO_2_), representing relative reductions of 40.13%, 67.97%, 43.59%, and 49.91%, respectively, which enhances electrical conductivity and charge transport efficiency;
2. GaNNTs modified with different metal oxides display distinct CF_3_CN adsorption characteristics, with CuO-GaNNT, Ag_2_O-GaNNT, and CrO_2_-GaNNT predominantly undergoing physisorption while ZnO-GaNNT demonstrates chemisorption features, and the adsorption strengths follow the order: ZnO-GaNNT > Ag_2_O-GaNNT > CuO-GaNNT > CrO_2_-GaNNT;
3. at room temperature, CF_3_CN desorbs relatively quickly from Ag_2_O-GaNNT (4.09 s), CuO-GaNNT (5.57 μs), and CrO_2_-GaNNT (1.28 × 10^−2^ μs), enabling rapid gas detection, whereas ZnO-GaNNT exhibits a significantly longer theoretical recovery time, making it more suitable for sustained CF_3_CN capture.

In conclusion, surface modification of GaNNTs with metal oxides (CuO, ZnO, Ag_2_O, CrO_2_) provides a highly effective approach for CF_3_CN detection and adsorption, identifying new avenues for environmental monitoring while offering a theoretical basis for optimizing the eco-friendly operation of high-voltage electrical equipment, such as gas-insulated circuit breakers and transformers, and enhancing condition monitoring strategies.

## Data Availability

The original contributions presented in the study are included in the article/supplementary material, further inquiries can be directed to the corresponding author.
